# Synthesis, Characterization, and Biological Studies of Organotin(IV) Derivatives with o- or p-hydroxybenzoic Acids

**DOI:** 10.1155/2009/542979

**Published:** 2009-04-15

**Authors:** Mohamed A. Abdellah, Sotiris K. Hadjikakou, Nick Hadjiliadis, Maciej Kubicki, Thomas Bakas, Nikolaos Kourkoumelis, Yannis V. Simos, Spyros Karkabounas, Mirela M. Barsan, Ian S. Butler

**Affiliations:** ^1^Section of Inorganic and Analytical Chemistry, Department of Chemistry, University of Ioannina, 45110 Ioannina, Greece; ^2^Department of Chemistry, Qena Faculty of Science, South Valley University, Qena 83523, Egypt; ^3^Faculty of Chemistry, Adam Mickiewicz University, ul. Grunwaldzka 6, 60-780 Poznan, Poland; ^4^Physics of Material Laboratory, Department of Physics, University of Ioannina, 45110 Ioannina, Greece; ^5^Medical Physics Laboratory, Medical School, University of Ioannina, 45110 Ioannina, Greece; ^6^Department of Experimental Physiology, Medical School, University of Ioannina, 45110 Ioannina, Greece; ^7^Department of Chemistry, McGill University, 801 Sherbrooke, Montreal QC, Canada H2A 2K6

## Abstract

Organotin(IV) complexes with o- or p-hydroxybenzoic acids (o-H_2_BZA or p-H_2_BZA) of formulae [R_2_Sn(HL)_2_] (where H_2_L = o-H_2_BZA and R = Me- (**1**), *n*-Bu- (**2**)); [R_3_Sn(HL)] (where H_2_L = o-H_2_BZA and R = *n*-Bu- (**3**), Ph- (**4**) or H_2_L = p-H_2_BZA and R = *n*-Bu- (**5**), Ph- (**6**)) were synthesized by reacting a methanolic solution of di- and triorganotin(IV) compounds with an aqueous solution of the ligand (o-H_2_BZA or p-H_2_BZA) containing equimolar amounts of potassium hydroxide. The complexes were characterized by elemental analysis, FT-IR, Far-IR, TGA-DTA, FT-Raman, Mössbauer spectroscopy, ^1^H, ^119^Sn-NMR, UV/Vis spectroscopy, and Mass spectroscopy. The X-ray crystal structures of complexes **1** and **2** have also been determined. Finally, the influence of these complexes **1–6** upon the catalytic peroxidation of linoleic acid to hydroperoxylinoleic acid by the enzyme lipoxygenase (LOX) was kinetically studied and the results showed that triorganotin(IV) complex **6** has the lowest IC_50_ value. Also complexes **1–6** were studied for their in vitro cytotoxicity against sarcoma cancer cells (mesenchymal tissue) from the Wistar rat, and the results showed that the complexes have high activity against these cell lines with triphenyltin((IV) complex **4** to be the most active one.

## 1. Introduction

Organotin compounds have many important applications and uses [1, 2]. Commercially, organotin compounds are used as industrial and agricultural biocides because they have high antifungal properties [3, 4]. The in vitro fungicidal or antibacterial properties of organotins have been found to exhibit the general order of activity: RSnX_3_ < R_2_SnX_2_ < R_4_Sn ≪ R_3_SnX, with the anionic X group to exert little influence on activity [5, 6]. The combination of two biologically active entities, however, in the same molecule could enhance their activity [7]. For example, triphenyltin(IV) derivatives of phthalic acid and salicaldehyde have significant activity toward a range of fungi [8, 9]. Recently, interests in organotin(IV) carboxylates are increasing due to their possible medical uses as antitumor agents [10]. For example, the fluoro-substituted carboxylate ligands with di- and triorganotins produced several antitumor active compounds [11]. Hubert et al. concluded that antitumor active tin compounds possess available coordination positions around tin atom and also have relatively stable ligand-tin bonds with low hydrolytic decomposition [12]. Thioamides-organotin complexes, on the other hand, have shown high antitumor activity, which is rather related to the ligand type and not to the geometry of the compounds [13–17]. Given that the antitumor action of Sn(IV) compounds may not be due to their direct interaction with DNA constituents [18–22], their reaction with enzymes like lipoxygenase is always of interest in the attempt to elucidate their mechanism of action [13–17]. This antitumor activity of the organotin complexes follows the same order of lipoxygenase inhibition, an enzyme taking part in the inflammation mechanism and tumor genesis [13–17].

**Scheme 1:**
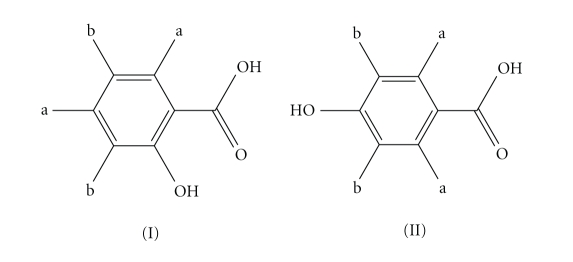


With the aim to prepare new organotin(IV-)-based antitumor compounds of o- or p-hydroxybenzoic acid (Schemes 1 (I) and (II), resp.), the synthesis of six organotin complexes of formulae [R_2_Sn(HL)_2_] (where H_2_L = o-H_2_BZA and R = Me- (**1**), *n*-Bu- (**2**)) and [R_3_Sn(HL)], (where H_2_L = o-H_2_BZA and R = *n*-Bu- (**3**), Ph- (**4**) or H_2_L = p-H_2_BZA, and R = *n*-Bu- (**5**), Ph- (**6**)) and their characterization by spectroscopic techniques (IR, Raman,^1^H-NMR, mass Spectra, ^119m^Sn Mössbauer, UV/Vis), elemental analysis and X-ray diffraction have been carried out. The inhibition caused by these complexes toward the oxidation of linoleic acid to hyperoxolinoleic acid by the enzyme lipoxygenase (LOX) follow the order **6** > **4** > **5** > **2** > **1** > **3** and compared with their in vitro antitumor activity against sarcoma cells, where the order is **4** > **6** > **5** ≫ **3** = **2** ≫ **1**. Therefore, triorganotin compounds inhibit stronger lipoxygenase and show higher activity against sarcoma cells than diorganotins.

## 2. Results and Discussion

### 2.1. General Aspects

Organotin(IV) complexes **1–6** have been synthesized by reacting a methanolic solution of organotin chloride with an aqueous solution of the appropriate amounts of 2- or 4-hydroxybenzoic acid containing an equimolar amount of potassium hydroxide as shown in (1) and (2):

(1) $$\begin{array}{l} {\text{R}}_{2}\text{SnC}{\text{l}}_{2}+2{\text{H}}_{2}\text{L}+2\text{KOH}\overset{\text{MeOH}/{\text{H}}_{2}\text{O}}{\to }\\ {\text{R}}_{2}\text{Sn(H}{\text{L}}_{2})+2\text{KCl}+2{\text{H}}_{2}\text{O}\\ {\text{H}}_{2}\text{L}={\text{o-H}}_{2}\text{BZA}\;\;\text{and}\;\;\text{R}=\text{Me-}\;\;(1),\;\;\text{n-Bu-}\;\;(2) \end{array}$$

(2) $$\begin{array}{l} {\text{R}}_{3}\text{SnC}\text{l}+{\text{H}}_{2}\text{L}+\text{KOH}\overset{\text{MeOH}/{\text{H}}_{2}\text{O}}{\to }\\ {\text{R}}_{3}\text{Sn(HL})+\text{KCl}+{\text{H}}_{2}\text{O}\\ {\text{H}}_{2}\text{L}={\text{o-H}}_{2}\text{BZA}\;\;\text{and}\;\;\text{R}=\text{n-Bu- }\;(3),\;\text{ Ph- }\;(4)\;\;\text{or}\\ {\text{H}}_{2}\text{L}={\text{p-H}}_{2}\text{BZA}\;\;\text{and}\;\;\text{R}=\text{n-Bu- }\;(5),\;\text{ Ph- }\;(6). \end{array}$$

Complexes **1**-**2** were prepared with an alternative method of the one used previously for the synthesis of these complexes [23, 24]. Complexes **1–6** are air-stable powders soluble in methanol, ethanol, and DMSO solvents. Crystals suitable for X-ray analysis were obtained by slow evaporation of methanol/acetonitril solutions for compounds **1** and **2**.

### 2.2. Thermal Analysis

**Scheme 2:**
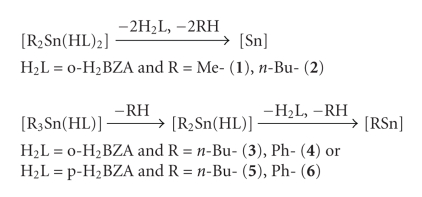


The TGA/DTA data curves for complexes **1**-**2** show that they decompose generally in one stage. Thus, thermal analysis in flowing nitrogen shows that complex **1** decomposes between 125 and 315°C with 72% mass loss which corresponds to the methyl groups of the metal and the ligand molecules (the calculated mass loss is 72%), compound **2** decomposes between 35 and 490°C with 74% mass loss due to the butyl groups of the metal and the ligand molecules (the calculated mass loss is 76.50%) (Scheme 2).

The TGA/DTA data in flowing nitrogen data curves for complexes **3–6** show that they decompose generally in two stages. The first stage of decomposition of compound **3** lies between 20 and 255°C that corresponds to 13% mass loss of one of the metal butyl groups (the calculated mass loss is 13%), compound **4** decomposes between 35 and 215°C with 16% mass loss of one of the metal phenyl groups (the calculated mass loss is 16%), compound **5** decomposes between 25 and 255°C that corresponds to 13% mass loss of one of the metal butyl groups (the calculated mass loss is 13%), and compound **6** decomposes between 25 and 177°C with 15% mass loss of one of the metal phenyl groups (the calculated mass loss is 16%). The second stage of decomposition is between 255 and 400°C in case of **3** which corresponds to 44% mass loss of the ligand (o-hydroxybenzoic acid) and another metal butyl group (the calculated mass loss is 45%), between 215 and 500°C (**4**) corresponding to 43% mass loss of the ligand (o-hydroxybenzoic acid) and another metal phenyl group (the calculated mass loss is 42%), compound **5** decomposes between 255 and 412°C which corresponds to 45.60% mass loss of the ligand (p-hydroxybenzoic acid) and another metal butyl group (the calculated mass loss is 45%), and compound **6** decomposes between 177 and 435°C with 42% mass loss which corresponds to the ligand (p-hydroxybenzoic acid) and another metal phenyl group (the calculated mass loss is 42%) (Scheme 2).

### 2.3. Spectroscopy

*Vibrational spectroscopy:* characteristic infrared bands of the complexes **1–6** and the ligands are listed in Table 1.

The IR spectra of the complexes **1–6** show vibrational bands at 3467 **1**, 3450 **2**, 3450 **3**, 3447 **4**, 3439 **5**, and 3433 **6** cm^−1^, which are assigned to *v*(phenolic OH) [28, 29]. The corresponding vibrational bands *v*(O-H) of the free ligands appear at 3282 and 3382 cm^−1^ for o-H_2_BZA or p-H_2_BZA, respectively [25, 27].

The *v*_as_(COO^−^) vibration of the free ligand appears at 1656 cm^−1^ while the *v*_s_(COO^−^) at 1324 cm^−1^ for o-H_2_BZA [25], and the *v*_as_(COO^−^) vibration of p-H_2_BZA ligand appears at 1687 cm^−1^ while the *v*_s_(COO^−^) at 1360 cm^−1^ [27]. The corresponding *v*_as_(COO^−^) vibrations are observed at 1631 **1**, 1628 **2**, 1633 **3**, 1636 **4**, 1636 **5**, and 1617 **6** cm^−1^, respectively. The bands at 1388 **1**, 1419 **2**, 1352 **3**, 1356 **4**, 1419 **5**, and 1347 **6** cm^−1^ are assigned to *v*_s_(COO^−^). The Δ*v* [*v*_as_(COO^−^)-*v*_s_(COO^−^)] difference values of the free ligands are (332 cm^−1^ for o-H_2_BZA and 327 cm^−1^ for p-H_2_BZA) versus the corresponding values Δ*v* for complexes **1–6** (243 **1**, 209 **2**, 281 **3**, 280 **4**, 217 **5**, and 270 **6** cm^−1^, resp.) support the coordination of the ligand to the metal center through the carboxylic acid group. Monodentate coordination of the –COO^−^ group of the ligand to metal ions results in significantly higher Δ*v* [*v*_as_(COO^−^)-*v*_s_(COO^−^)] difference values than those observed for the ionic compounds of the ligand [30], while when the ligand chelates, the Δ*v* [*v*_as_(COO^−^)-*v*_s_(COO^−^)] is considerably smaller than that observed for its ionic compounds. For asymmetric bidentate coordination, the values are in the range of monodentate coordination [30]. When the –COO^−^ group bridges metal ions, the Δ*v* [*v*_as_(COO^−^)-*v*_s_(COO^−^)] value is higher than that of the chelated ions and nearly the same as that observed for ionic compounds [30]. In our case, the Δ*v* [*v*_as_(COO^−^)-*v*_s_(COO^−^)] values of the ionic compounds of the o-NaHBZA and p-NaHBZA ligands (sodium salts) are 205 cm^−1^ and 131 cm^−1^, respectively [26]. Therefore, in the diorganotin complexes **1** and **2**, where higher Δ*v* values were observed (243 **1** and 209 **2** cm^−1^), an asymmetric bidentate coordination of the ligand to the metal ion is expected (see crystal structure, Mössbauer spectra).The same is true in the case of triorganotin complexes **3–6** with significantly higher Δ*v* values than the corresponding ones of the sodium salts of the ligands (281 **3**, 280 **4**, 217 **5**, and 270 **6** cm^−1^), indicating clearly an asymmetric bidentate coordination mode of the ligands and suggesting trigonal bipyramidal geometry for complexes **3–6** in the solid state in agreement with the results of Mössbauer and ^119^Sn-NMR spectra.

Bands at 425–455 cm^−1^ have been assigned to the stretching vibration of Sn-O bonds. The corresponding ones for complexes **1–6** are at 446 **1**, 435 **2**, 445 **3**, 420 **4**, 458 **5**, and 450 cm^−1^ **6** [25]. Bands at 600–500 cm^−1^ have been assigned to the antisymmetric and symmetric vibrations of Sn-C bond. The corresponding ones for complexes **1–6** are at 580, 536 **1**, 562, 530 **2**, 564, 538 **3**, 600, 533 **4**, 610, 509 **5**, and 569, 511 **6** [13–17]. No *ν*(Sn-Cl) vibration bands at 282 and 221 cm^−1^ are observed in the far FT-IR of the complexes [13].

^119m^Sn *Mössbauer spectroscopy:* solid state, ^119m^Sn Mössbauer spectroscopic data of complexes **1**-**2** and **4–6** are given in Table 2.

The isomer shifts values of complexes **1–6** are in the range of *δ* 1.22 to 1.56 mm s^−1^ (Table 2), indicating that tin is in the (4+) oxidation state in all cases [2, 32, 33]. The spectra of diorganotin(IV) complexes **1–2** consist of two symmetrical Lorentzian doublets which indicate the presence of two tin atoms in different chemical environments with the same ratio 1 : 1 (56 : 44% **1** and 29 : 71% **2**, resp.). This may be due to the presence of two different isomers in the unit cells with variable bond distances. The quadrupole splitting values (Δ) of complexes **1**-**2** are 3.09 mm s^−1^ and 3.65 mm s^−1^ for **1** and 3.53 mm s^−1^ and 3.68 mm s^−1^ for **2**, suggesting distorted *trans*-R_2_ octahedral geometry (2.4–5.5 mm s^−1^) [2, 32, 33] in the solid state in agreement with X-ray structures. The calculated C-Sn-C angles (°) from Mössbauer spectra [31] for the compounds **1** and **2** (Table 2) are 130° for the highest occurring isomer (56%) and 143° for the lowest occurring isomer (44%) in case of **1** and 148 (29%), 149° (71%) in case of **2**. The values of C-Sn-C angles found from X-ray analysis at 293 K are 138.4(4)° (**1**) and 144.0(3)° (**2**), respectively (see crystal structures).

The spectra of the triorganotin(IV) complexes **4–6** consist of one symmetrical Lorentzian doublet which indicates the presence of one tin atom. The quadrupole splitting values in case of complexes **4–6** (Δ) are 2.93 mm s^−1^ **4**, 2.48 mm s^−1^ for **5**, and 3.18 mm s^−1^ **6** suggesting eq-R_3_ trigonal bipyramidal geometry around tin atom (2.5–4.0 mm s^−1^) [2, 32, 33] in solid state in agreement with the geometry concluded from ^119^Sn-NMR spectra.

### 2.4. NMR Spectra

^1^H-NMR spectra of complexes **1–6** showed a single resonance signal at 10.18 **1**, 10.16 **2**, 9.91 **3**, 9.95 **4**, 9.87 **5**, and 9.89 **6** ppm, respectively, due to the hydroxyl proton of the ligands (o-H_2_BZA or p-H_2_BZA) [34], suggesting that the OH groups are not involved in bonding with the tin atom. The two doublet signals at 7.83-7.79 and 6.82-6.79 **1**, 7.69-7.65 and 6.75-6.72 **2**, 7.72-7.69 and 6.73-6.70 **3**, 7.81-7.78 and 6.69-6.65 **4**, and 7.75-7.71 and 6.76-6.73 **6** ppm are assigned to the protons of the phenyl group of the ligands *a* and *b*, respectively (Scheme 1) [34]. In case of complex **1**, the single signal at 0.83 ppm is assigned to the protons of the methyl group of the metal.

In case of complexes **6** and **7**, ^119^Sn-NMR spectra were also recorded. ^119^Sn-NMR spectra showed resonance signals at −20.0 (**6**) and −212.0 (**7**) ppm, respectively. Although the shift ranges are somewhat dependent on the nature of the substituents at the tin atom the *δ*(^119^Sn) values (in ppm) for R_3_Sn(IV) complexes with five-coordinated Sn vary from +25 to −329 ppm [33]. Thus, the tin(IV) atoms are five coordinated in case of complexes **5** and **6**, and therefore the expected geometry arrangement around triorganotin(IV), in solution, is suggested as trigonal bipyramidal.

**Crystal, Molecular Structures of [(CH_3_)_2_Sn(OOCC_6_H_4_OH)_2_] (1) and [(n-C_4_H_9_)_2_Sn(OOCC_6_H_4_OH)_2_] (2)**

**Figure 1:**
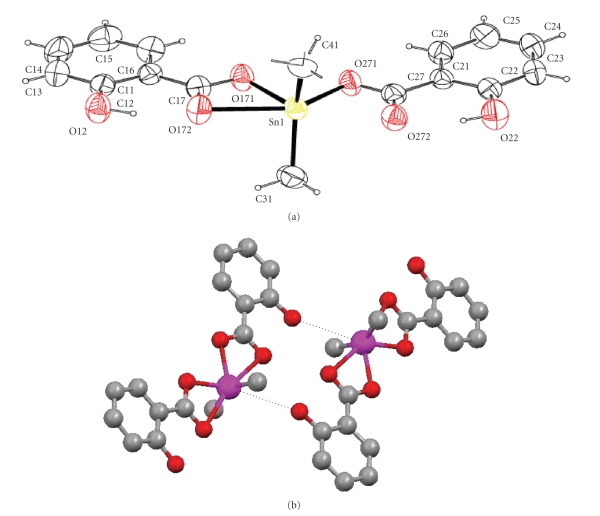
(a) Anisotropic ellipsoid representation of complex **1**. The ellipsoids are drawn at 50% probability level. Selected bond distances (Å) and bond angles (deg.): Sn1-O171 = 2.1069(18), Sn1-O172 = 2.5145(15), Sn1-O271 = 2.1086(15), Sn1-O272 = 2.577, Sn1-C31 = 2.086(3), Sn1-C41 = 2.091(3), O171-Sn1-O172 = 55.56(5), O172-Sn1-O272 = 83.39(6), and O271-Sn1-O171-C17 = 178.46(14). (b) Strong Sn ⋯ O bonding interaction leads to the formation of a dimer.

**Figure 2:**
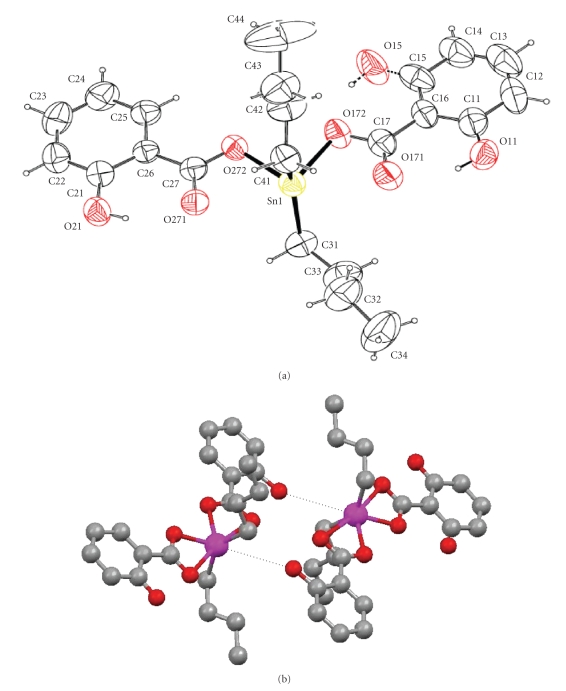
(a) Anisotropic ellipsoid representation of complex **2**. The ellipsoids are drawn at 50% probability level. Selected bond distances (Å) and bond angles (deg.): Sn1-O172 = 2.121(3), Sn1-O171 = 2.565, Sn1-O272 = 2.105(4), Sn1-O271 = 2.632, Sn1-C31 = 2.120(6), Sn1-C41 = 2.111(5), O172-Sn1-O272 = 81.66(13), O171-Sn1-O271 = 170.01, and O272-Sn1-O172-C17 = 177.6(4)°. (b) Strong Sn ⋯ O bonding interaction leads to the formation of a dimer.

The crystal structures of complexes **1** and **2** had been determined previously, at 193 and 295 K with R% values of 6.4 and 3.8, respectively [23, 24]. However, we redetermined the structures here of both **1** or **2** complexes at room temperature (293 K), with R% 2.18 and 4.83, respectively, for comparison and in order to investigate the influence of the temperature on their structures [33]. ORTEP diagrams of complexes **1** and **2** are shown in Figures 1 and 2.

Compounds **1** and **2** are covalent monomers in the solid state with a distorted octahedral geometry around the metal ion. Table 3 summarizes Sn-O and other selected bond distances and angles found for organotin complexes reported here and elsewhere [23, 24]. They differ only slightly.

The Sn-O bond distances are 2.1060(18), 2.5147(15), 2.1079(15), and 2.577 Å in **1**; 2.104(4), 2.564, 2.121(4), and 2.632 Å in **2** showing an asymmetric bidentate coordination of the ligand to the Sn atom, as concluded in the IR Section 2.3. The Sn-O bond distances found in **1** and **2** are in agreement with the corresponding bond lengths found in (CH_3_)_2_Sn(2, 4, 5-TF-3-MBA)_2_ (2,4,5-TF-3-MBA = 2,4,5-trifluoro-3-methoxybenzoic acid) 2.115(6)–2.656(6) Å [11], in (*n*-C_4_H_9_)_2_Sn(2, 4, 5-TF-3-MBA)_2_, 2.128(3)–2.562(3) Å [11], and in (*n*-C_4_H_9_)_2_Sn(2, 4-DHB)_2_ (2,4-DHB = dihydroxybenzoato) 2.110(4)–2.559(4) Å [35].

The O-Sn-O-C torsion angles found in complexes **1** and **2** were O271-Sn1-O171-C17 = 178.46(14)° for **1** and (O272-Sn1-O172-C17 = 177.6(4)° for **2**. This indicates the almost coplanar arrangement of the two ligands with the tin atom. Thus, the conformation around the tin atom is *trans*-C_2_, *cis*-O_2_, *cis *O_2_. The value of the C-Sn-C angle (C31-Sn1-C41 = 138.44(11)° in **1** and C31-Sn1-C41 = 143.8(2)° in **2**) implies distortion of the octahedral structure.

In both complexes **1** and **2** there are intramolecular hydrogen bonding interactions (O12 ⋯ O172 = 2.620(2) Å and O22 ⋯ O272 = 2.605(2) Å for **1**, O11 ⋯ O171 = 2.621(7) Å and O21 ⋯ O271 = 2.622(5) Å in case of **2**) stabilizing the structures. Interesting strong Sn ⋯ O bonding interaction (Sn1 ⋯ O12 = 3.463 Å in case of **1** and Sn1 ⋯ O21 = 3.620 Å in case of **2**) leads to the formation of dimers in both cases (Figures 1 and 2) which may affect the distortion of the octahedron observed. The sum of van der Walls radii of Sn and O varied between 3.80 and 4.17 Å [36]. No such interaction was observed earlier [23, 24].

**Study of the Peroxidation of Linoleic Acid by the Enzyme Lipoxygenase in the Presence of Complexes 1–6**

The influence of complexes **1–6** on the oxidation of linoleic acid by the enzyme (LOX) was studied in a wide concentration range. The degree of (LOX) activity (A, %) in the presence of these complexes was calculated according to the method described previously [13]. The IC_50_ values found for complexes **1–7** are 76 (**1**), 48 (**2**), 82 (**3**), 19 (**4**), 24 (**5**), and 11 (**6**) *μ*m, respectively. Thus, five coordinated (see Sn-NMR data) triphenyl organotin compounds **6** and **4** are the most active among this type of complexes showing the order of activity: **6** > **4** > **5** > **2** > **1** > **3**. Moreover, triorganotin complexes with p-H_2_BZA are found to be more active than the corresponding compounds with p-H_2_BZA. These values are also comparable to the ones found for other organotin(IV) complexes [13–17] and significantly higher than the corresponding cisplatin. For example, the IC_50_ values found for the organotin compounds tested toward (LOX) were 25 *μ*m [(*n*-Bu)_3_Sn(TBA) · H_2_O] (HTBA = 2-thiobarbituric acid) [14], 26 and 14 *μ*m for [(C_6_H_5_)_2_SnCl(HMNA)] and [(C_6_H_5_)_3_Sn(MNA)Sn(C_6_H_5_)_3_(acetone)] (H_2_MNA = 2-mercapto-nicotinic acid) [13, 14], 19, 16, and 21 *μ*m for [(C_6_H_5_)_3_Sn(MBZT)], [(C_6_H_5_)_3_Sn(MBZO)], [(C_6_H_5_)_3_Sn(CMBZT)] [15] (MBZT = 2-mercapto-benzothiazole, MBZO = 2-mercapto-benzoxazole and CMBZT = 5-chloro-2-mercapto-benzothiazole), 10, 13, and 14 *μ*m for [(C_6_H_5_)_2_Sn(CMBZT)_2_], [(*n*-C_4_H_9_)_2_Sn(CMBZT)_2_], and [(CH_3_)_2_Sn(CMBZT)_2_] [15], and 61.3, 26.2, 20.5, and 16.9 *μ*m for [(CH_3_)_2_Sn(PMT)_2_], [(*n*-C_4_H_9_)_2_Sn(PMT)_2_], [(C_6_H_5_)_2_Sn(PMT)_2_], and [(C_6_H_5_)_3_Sn(PMT)] (PMT = 2-mercapto-pyrimidine) [16]. Taking into account that higher LOX inhibition activity is found to be related to the high cytotoxic activity against sarcoma cells, compound **4, 5,** and **6** are expected to show such high activity (see below).

### 2.5. Biological Tests

The high inhibitory activities of complexes **1–10** against LOX activity found prompted us to study the antiproliferative activity of them against sarcoma cells and compare these data with their structural features. Complexes **1–6** were tested for cytotoxic activity against leiomyosarcoma cells from the Wistar rat, polycyclic aromatic hydrocarbons (PAH, benzo[a]pyrene) carcinogenesis. Cytotoxic activities for complexes **1–6** were evaluated as % percentage of the cell survived in variable concentrations of the complexes after 48 hours. The IC_50_ values found were >2000 (**1**), 150 (**2**), 150 (**3**), 5–10 (**4**), 30–40 (**5),** and 25–35 (**6**) nm, respectively, indicating very strong cytotoxic activity against leiomyosarcoma cells. In accordance with the LOX inhibition activity, the five-coordinated triorganotin complexes **5, 3,** and **6** showed stronger antiproliferative activity against sarcoma cell lines (see LOX inhibition activity). The order of activity of complexes **1–6** is found to be **4** > **6** > **5** ≫ **3** = **2** ≫ **1**. It is noteworthy to mention that triorganotin(IV) compounds **4–6** were found to inhibit LOX activity and anti-proliferate sarcoma cells stronger than di-organotins. Between diorganotin(IV) derivatives of o-H_2_BZA **1** and **2**, the dibutyltin complex exhibits significant stronger cytotoxic activity than the corresponding one of dimethyltin complex. This is also observed in the case of substituted salicylic acids reported previously [34], where diethyl derivatives were found to show approximately 10 times lower activity than the corresponding dibutyltin complexes against human mammary tumor cell lines (MCF-7) and human colon carcinoma cell line (WiDr) [34]. The corresponding IC_50_ values of other organotin(IV) complexes found against leiomyosarcoma cells were 5 and 125 nm for [(C_6_H_5_)_3_Sn(MNA)Sn(C_6_H_5_)_3_(acetone)] (H_2_MNA = 2-mercapto-nicotinic acid) [14], and [(*n*-Bu)_3_Sn(TBA) · H_2_O] (HTBA = 2-thiobarbituric acid) [14], 1500–3000, 1300–3000, and 500–800 nm for [(C_6_H_5_)_3_Sn(MBZT)], [(C_6_H_5_)_3_Sn(MBZO)], [(C_6_H_5_)_3_Sn(CMBZT)] (MBZT = 2-mercapto-benzothiazole, MBZO = 2-mercapto-benzoxazole and CMBZT = 5-chloro-2-mercapto-benzothiazole) [15], 300–500, 600–800, and 5000–7500 nm for [(C_6_H_5_)_2_Sn(CMBZT)_2_], [(*n*-C_4_H_9_)_2_Sn(CMBZT)_2_], and [(CH_3_)_2_Sn(CMBZT)_2_], [15] and 20000–60000, 700, 1000–2000, and 100 nm for [(CH_3_)_2_Sn(PMT)_2_], [(*n*-C_4_H_9_)_2_Sn(PMT)_2_], [(C_6_H_5_)_2_Sn(PMT)_2_], and [(C_6_H_5_)_3_Sn(PMT)] (PMT = 2-mercapto-pyrimidine), [16]. From all of these values for the different compounds, it is clear that complexes **1–6** have high cytotoxic activity against leiomyosarcoma cells. Triphenylorganotin(IV) complexes **4** and **6** are the most active compounds of this series of compounds (where the IC_50_ are 5–10 nm for **4** and 25–35 nm for **6**) and they are classified among the most active organotin(IV) compounds tested.

Table 4 summarizes the IC_50_ (*μ*m) values for LOX inhibition in comparison with cell activity of organotin(IV) complexes **1–6** against sarcoma cells.

### 2.6. Computational Methods–Docking Study

In order to investigate further the complex-LOX interactions, we performed computational molecular docking studies for the complexes **1** and **2** were X-ray data are available. The binding energy (*E*) of the substrate (S: linoleic acid) to its binding site in the enzyme LOX (**E**) when **ES** is the complex formed was *E:* −7.89 kcal/mole [13]. The corresponding binding energies of inhibitors **1** and **2** (I), in ESI, are calculated to −8.48 (**1**) and −8.23 (**2**) kcal/mol, respectively, while the binding energies of EI are estimated to −9.7 Kcal/mol (**1**) and −11.4 (**2**) kcal/mol. According to the binding energy (*E*) values of ES in contrast to those of EI or ESI, it is found that both ESI and EI complexes could be formed.

**Figure 3:**
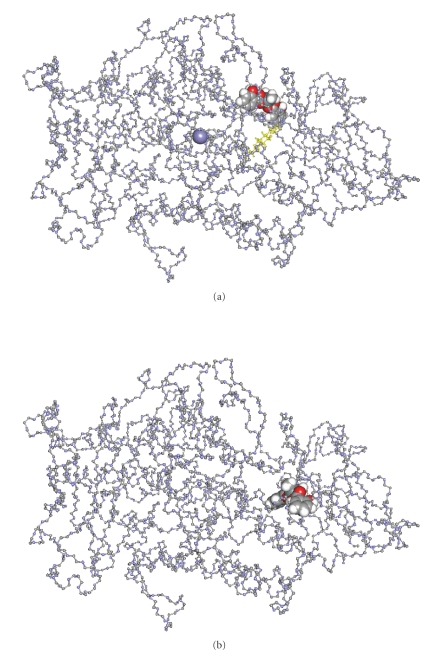
Binding sites of inhibitor **1** toward LOX in **ESI** and **EI,** respectively.

**Figure 4:**
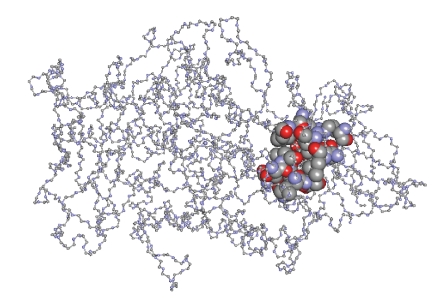


Figures 3 and 4 show the binding site of compound **1** toward LOX in **ESI** and **EI,** respectively. Compounds **1** and **2** bind to both **ESI** and **EI** complexes at the same pocket where the strong inhibitors of LOX bind [15], supporting its strong inhibition activity, found experimentally. Since high inhibition activity of LOX has been detected for all cytotoxic organotin(IV)-thione compounds tested previously, [13–17] strong activity would also be expected for compounds **1** and **2,** although weaker than the others, tested in this study.

## 3. Conclusion

Organotin(IV) complexes (**1**)–(**7**) are found to inhibit strongly the peroxidation of linoleic acid by the enzyme lipoxygenase. Five-coordinated organotin(IV) complexes **6**, **4,** and **5** (IC_50_ = 11, 19, and 24 *μ*m, resp.) showed strongest LOX inhibition activity than the corresponding six-coordinated one (Table 4). Compounds **1–6**, also, showed strong antitumor activity against sarcoma cells (Table 4). The highest antiproliferate activity against sarcoma cell lines is also shown by the five-coordinated triphenyltin(IV) complexes **4** and **6** (IC_50_ = 5–10 and 25–35 nm, resp.). Between tri- and diorganotin complexes, five-coordinated organotin complexes which have one free coordination position were found to exhibit stronger antiproliferative and LOX inhibition activity. These findings are in accordance to Huber et al. [12] who suggested that the structures of organotin carboxylates antitumor active compounds are characterized by (i) the availability of coordination positions at Sn and (ii) the occurrence of relatively stable ligand-Sn bonds, for example, Sn-N and Sn-S and their slow hydrolytic decomposition. Therefore, the geometrical feature of this type of complexes (**1–6**) seems to play important role in their antitumor and LOX inhibition activity.

## 4. Experimental

### 4.1. Materials and Instruments

All solvents used were of reagent grade, while o-, p-hydroxybenzoic acid, and organotins chlorides (Alderich, USA), (Merck, Germany) were used with no further purification. Elemental analysis for C and H was carried out with a Carlo Erba EA MODEL 1108. Infrared spectra in the region of 4000-370 cm^−1^ were obtained in KBr discs. The ^119^Sn Mössbauer spectra were collected at 80 K, with a constant acceleration spectrophotometer equipped with CaSnO_3_source kept at low temperature. A Jasco UV/Vis/NIR V570 series spectrophotometer was used to obtain electronic absorption spectra. The ^1^H-NMR spectra were recorded on a Bruker AC250 MHFT NMR instrument in DMSO-*d*_6_ solution. Chemical shifts *δ* are reported in ppm using ^1^H TMS as an internal reference.

**Preparation of the Complexes [(CH_3_)_2_Sn(o-HBZA)_2_] (1), [(*n*-C_4_H_9_)_2_Sn(o-HBZA)_2_] (2), [(*n*-C_4_H_9_)_3_Sn(o-HBZA)_2_] (3), [(C_6_H_5_)_3_Sn(o-HBZA)_2_] (4), [(*n*-C_4_H_9_)_3_Sn(p-HBZA)_2_] (5), and [(C_6_H_5_)_3_Sn(p-HBZA)_2_] (6).**

Complexes **1**-**2** were synthesized as follows: a suspension of the ligand (o-hydroxybenzoic acid, 0.138 gr, 1 mmol **1**-**2**) in 5 cm^−3^ distilled water was treated with a solution of KOH 1 N (1 cm^−3^, 1 mmol **1**-**2**) and a clear solution was immediately formed. 5 cm^−3^ methanolic solution of diorganotin(IV) chloride ((CH_3_)_2_SnCl_2_, 0.109 gr, 0.5 mmol for **1** and (*n*-C_4_H_9_)_2_SnCl_2_, 0.152 gr, 0.5 mmol for **2**) was then added to the above solution. A white precipitate formed and the mixture was stirred for 4 hours. The precipitate was then filtered off, washed with 3 mL of distilled water and dried in vacuo over silica gel. Crystals of **1** and **2** complexes suitable for X-ray analysis were growth by slow evaporation of CH_3_OH/CH_3_CN solutions (1 : 1). Complexes **3–6** were synthesized as follows: a suspension of the ligand (o-, p-hydroxybenzoic acid, 0.069 gr, 0.5 mmol **3–6**) in 5 cm^−3^ distilled water was treated with a solution of KOH 1 N (0.5 cm^−3^, 0.5 mmol **3–6**) and a clear solution was immediately formed. 5 cm^−3^ methanolic solution of triorganotin(IV) chloride ((*n*-C_4_H_9_)_3_SnCl, 135.6 *μ*L, 0.5 mmol for **3** and **5**, (C_6_H_5_)_3_SnCl, 0.193 gr, 0.5 mmol for **4** and **6**) was then added to the above solution. A white precipitate formed and the mixture was stirred for 4 hours. The precipitate was then filtered off, washed with 3 mL of distilled water, and dried in vacuo over silica gel.

**1** Yield 70.25%, mp > 250°C. Elemental analysis found C 45.21, H 3.95%; calcd for C_16_H_16_O_6_Sn C 45.4, H 3.81%. IR (cm^−1^): 3467, 1631, 1444, 1388, 1157, 870, 700 580, 536, 446. ^1^H-NMR (DMSO-*d*_6_ , ppm): *δ* 10.18 (s, 1H, phenolic OH), 7.83, 6.82 (d, 4H, C_6_H_4_CO_2_OH), 0.83 (s, 6H, Sn-CH_3_).

**2** Yield 65%, mp 63°C. Elemental analysis found C 52.53, H 5.43%; calcd for C_22_H_28_O_6_Sn C 52.1, H 5.56%. IR (cm^−1^): 3450, 1628, 1419, 1334, 1159, 869, 702, 562, 530, 435. ^1^H-NMR (DMSO-*d*_6_, ppm): *δ* 10.16 (s, 1H, phenolic OH), 7.69, 6.75 (d, 4H, C_6_H_4_CO_2_OH), 0.86, 1.18, and 1.60 (t, m, 9H, Sn-C_4_H_9_).

**3** Yield 55.30%, mp > 250°C. Elemental analysis found C 53.95, H 7.42%; calcd for C_19_H_32_O_3_Sn C 53.43, H 7.55%. IR (cm^−1^): 3450, 1633, 1458, 1352, 1157, 866, 703, 564, 538, 445. ^1^H-NMR (DMSO-*d*_6_, ppm): *δ* 9.91 (s, 1H, phenolic OH), 7.78, 6.66 (d, 4H, C_6_H_4_CO_2_OH), 0.88, 1.20, and 1.62 (t, m, 9H, Sn-C_4_H_9_).

**4** Yield 54.35%, mp 160°C. Elemental analysis found C 61.78, H 3.98%; calcd for C_25_H_20_O_3_Sn C 61.64, H 4.14%. IR (cm^−1^): 3447, 1636, 1458, 1356, 1160, 863, 729, 600, 533, 420. ^1^H-NMR (DMSO-*d*_6_, ppm): *δ* 9.95 (s, 1H, phenolic OH), 7.72, 6.73 (d, 4H, C_6_H_4_CO_2_OH), 7.40–7.64 (m, 15H, Sn-C_6_H_5_).

**5** Yield 75.40%, mp > 285°C. Elemental analysis found C 53.66, H 8.25%; calcd for C_19_H_32_O_3_Sn C 53.43, H 7.55%. IR (cm^−1^): 3190, 1636, 1458, 1419, 1165, 854, 702, 610, 509, 458. ^1^H-NMR (DMSO-*d*_6_, ppm): *δ* 9.87 (s, 1H, phenolic OH), 7.81, 6.69 (d, 4H, C_6_H_4_CO_2_OH), 0.90, 1.29, and 1.69 (t, m, 9H, Sn-C_4_H_9_). ^119^Sn-NMR: -20. UV-Vis (solvent) *λ*_max_ (*ε*): (DMSO) 237 (11418), (CHCl_3_) 228 (9796) and (MeOH) 227 (15576). ESI-MS (MeOH, m/z): 450.0 [{(*n*-C_4_H_9_)_3_Sn(p-HBZA)} + Na]^+^, 370.0 [(M)-(C_4_H_9_)]^+^, 336.0 [(M)-(C_4_H_9_)_2_]^+^, and 284.0 [{(M)-(C_4_H_9_)_3_} + Na]^+^.

**6** Yield 50.05%, mp 160°C. Elemental analysis found C 61.96, H 4.20%; calcd for C_25_H_20_O_3_Sn C 61.64, H 4.14%. IR (cm^−1^): 3234, 1617, 1431, 1347, 1166, 857, 695, 569, 511, 450. ^1^H-NMR (DMSO-*d*_6_, ppm): *δ* 9.89 (s, 1H, phenolic OH), 7.75, 6.76 (d, 4H, C_6_H_4_CO_2_OH), 7.42–7.66 (m, 15H, Sn-C_6_H_5_). ^119^Sn-NMR: -212. UV-Vis (solvent) *λ*_max_ (*ε*): (DMSO) 234 (5998), (CHCl_3_) 232 (13123) and (MeOH) 225 (9695) ESI-MS (MeOH, m/z): 510.0 [{(C_6_H_5_)_3_Sn(p-HBZA)} + Na]^+^ ions, 434.0 [{(M)-(C_6_H_5_)} + Na]^+^, and 356.0 [{(M)-(C_6_H_5_)_2_} + Na]^+^.

### 4.2. Study of the Lipoxygenase Inhibition and Biological Tests

Experimental details for LOX inhibition activity of complexes 1–6 and their in vitro cells toxicity studies were described earlier [13–15].

### 4.3. Computational Details

Computational details have been described elsewhere [13–15].

### 4.4. X-Ray Structure Determination

Data were collected by the *ω* scan technique in the range 4.86° < 2*θ* < 24.71° on a KUMA KM4CCD four-circle diffractometer [37] with CCD detector, using graphite-monochromated Mo K*α* (*λ* = 0.71073 Å) at 293(2) K. Cell parameters were determined by a least-squares fit [38]. All data were corrected for Lorentz-polarization effects and absorption [38, 39].

The structure was solved with direct methods with SHELXS97 [40] and refined by full-matrix least-squares procedures on *F* ^2^ with SHELXL97 [41]. All nonhydrogen atoms were refined anisotropically, hydrogen atoms were located at calculated positions and refined as a “riding model” with isotropic thermal parameters fixed at 1.2 times the *U*_eq_ of appropriate carrier atom.

**1. **C_16_H_16_O_6_Sn: MW = 423.00, monoclinic, P2_1_/*n*, *a* = 7.5321(3) Å, *b* = 21.8775(7) Å, *c* = 10.4440(4) Å, *β* = 105.505(4), *V* = 1658.37(11) Å, *Z* = 4, *T* = 293(1) K, *ρ*(cald) = 1.694 g cm^−3^, *μ* = 1.6 mm^−1^, *R* = 0.0218, w*R* = 0.0540.

**2. **C_22_H_26_O_6_Sn: MW = 505.14, monoclinic, P2_1_/*c*, *a* = 9.3545(16) Å, *b* = 24.135(4) Å, *c* = 10.8625(18) Å, *β* = 107.781(18), *V* = 2335.3(7) Å, *Z* = 4, *T* = 293(1) K, *ρ*(cald) = 1.440 g cm^−3^, *μ* = 1.1 mm^−1^, *R* = 0.0483, w*R* = 0.1007.

Crystallographic data (excluding structure factors) for the structures reported in this paper have been deposited with the Cambridge Crystallographic Data Centre as supplementary publication nos. CCDC 705791 (**1**) and CCDC 705792 (**2**). Copies of the data can be obtained free of charge on application to CCDC (Cambridge, UK).

## Figures and Tables

**Table 1:** Characteristic vibration bands (cm^−1^) of the o- or p-HBZA ligands and their complexes **1–6**.

Compound	Infrared					Raman		Ref.
	*v*(OH)	*v*(CH)	*v*_as_, *v*_sy_(COO)	*v*(Sn-C)	*v*(Sn-C)	*v*(Sn-O)	*v*(Sn-C)	
o-H_2_BZA	3282		1656, 1324					[25]
o-NaHBZA			1577, 1373					[26]
1	3467	2817-2705	1631, 1388	580, 536	446	—	—	
2	3450	2928-2869	1628, 1419	562, 530	435	—	—	
3	3450	2957-2364	1633, 1352	564, 538	445	—	—	
4	3447	3063	1636, 1356	600, 533	420	—	—	
p-H_2_BZA	3382	—	1687, 1360	—	—	—	—	[27]
p-NaHBZA	3382		1547, 1416					[27]
5	3439	2958-2922	1613, 1351	606, 509	457	410	520	
6	3433	3068	1615, 1431	569, 511	438	450	618	

**Table 2:** ^119^Sn Mössbauer spectroscopic data for complexes **1–6** at 80 K.

Molecule	*δ* (mm s^−1^)	Δ (mm s^−1^)	C-Sn-C (°) angles *	Area (%)	*δ* (mm s^−1^)	Δ (mm s^−1^)	C-Sn-C (°) angles *	Area (%)
1	1.22	3.09	130	56	1.33	3.65	148	44
2	1.31	3.53	143	29	1.56	3.68	149	71
4	1.34	2.93		100	—	—		—
5	1.24	2.48		100	—	—		—
6	1.44	3.18		100	—	—		—

*The C-Sn-C angles (*θ*) are calculated from the equation following Δ = 4{R}[1 − 3cos^2^ *θ·*sin^2^ *θ*]^1/2^, where the partial quadrupole splitting (p.q.s.), mm · s^−1^, used in the calculations was ([R]-[Cl])^oct^ = −1.03 (R = Me, Bu) [31].

**Table 3:** Selected bond distances (Å) and angles (deg) for the known diorganotin compounds with carboxylate ligands.

Complex	Temperature (K)	Sn-O (Å)	Sn-C (Å)	C-O (Å)	C-Sn-C (°)	O-Sn-O (°)	Ref.
**1**	293	2.1060(18)	2.089(3)	1.350(3)	138.4(4)	83.4(5)	[*]
		2.5147(15)	2.093(3)	1.347(3)		139.1(5)	
		2.1079(15)		1.293(2)		55.7(5)	
		2.577		1.252(3)			

**2**	293	2.104(4)	2.124(7)	1.288(6)	144.0(3)	81.68(15)	[*]
		2.564	2.110(7)	1.237(7)			
		2.121(4)		1.245(8)			
		2.632		1.284(8)			

(CH_3_)_2_Sn(o-HBZA)_2_	193	2.112(3)	2.092(5)	1.301(5)	138.2(2)	56.0(1)	[24]
		2.503(3)	2.092(5)	1.256(5)		82.9(1)	
		2.111(3)		1.292(5)		137.9(1)	
		2.577(3)		1.254(5)		138.9(1)	
						166.1(1)	
						55.0(1)	

(*n*-C_4_H_9_)_2_Sn(o-HBZA)_2_	298	2.117(5)	2.122(9)	1.28(1)	143.9(3)	54.5(2)	[23]
		2.570(6)	2.105(9)	1.233(8)		81.6(2)	
		2.095(5)		1.294(9)		135.7(2)	
		2.630(6)		1.246(8)		136.1(2)	
						169.7(1)	
						54.1(2)	

(*n*-C_4_H_9_)_2_Sn(2, 4, 5-DHB)_2_	298	2.559(4)	2.114(6)	1.267(6)	139.0(3)	54.9(1)	[35]
		2.110(4)	2.107(7)	1.281(6)		138.0(1)	
		2.508(4)		1.246(6)		82.8(1)	
		2.124(4)		1.294(7)		167.0(1)	
						137.7(1)	
						55.3(1)	

(CH_3_)_2_Sn(2, 4, 5-TF-3-MBA)_2_	298	2.115(6)	2.091(8)	1.262(9)	142.2(4)	135.8(2)	[11]
		2.656(6)	2.090(9)	1.234(10)		53.0(2)	
		2.118(5)		1.288(10)		171.17(18)	
		2.506(6)		1.229(10)		80.3(2)	
						55.6(2)	
						133.3(2)	

(*n*-C_4_H_9_)_2_Sn(2, 4, 5-TF-3-MBA)_2_	298	2.128(3)	2.109(5)	1.287(6)	147.5(2)	137.80(12)	[11]
		2.141(3)	2.107(5)	1.226(6)		54.58(12)	
		2.531(4)		1.281(6)		167.61(12)	
		2.562(3)		1.245(6)		82.60(13)	
						55.31(12)	
						137.08(12)	

[*]: This work; 2HB-O,O: 2-hydroxybenzoato-*O*, *O*′; 2,4-DHB: Dihydroxybenzoato; 2,4,5-TF-3-MBA: 2,4,5-Trifluoro-3-methoxybenzoic acid.

**Table 4:** IC_50_ values for LOX inhibition activity and cell cytotoxic activity of organotin(IV) complexes **1–6**.

Compound	IC_50_ for LOX inhibition	IC_50_ cell activity	Ref
[(CH_3_)_2_Sn(*o*-HBZA)_2_] (1)	76 *μ*m	>2000 nm	[*]
[(*n*-C_4_H_9_)_2_Sn(*o*-HBZA)_2_] (2)	48 *μ*m	150 nm	[*]
[(*n*-C_4_H_9_)_3_Sn(*o*-HBZA)] (3)	82 *μ*m	150 nm	[*]
[(C_6_H_5_)_3_Sn(*o*-HBZA)] (4)	19 *μ*m	5-10 nm	[*]
[(*n*-C_4_H_9_)_3_Sn(*p*-HBZA)] (5)	24 *μ*m	30-40 nm	[*]
[(C_6_H_5_)_3_Sn(*p*-HBZA)] (6)	11 *μ*m	25-35 nm	[*]
(*n*-Bu)_3_Sn(TBA) · H_2_O]	25 *μ*m	125 nM	[14]
[(C_6_H_5_)_3_Sn(MNA)Sn(C_6_H_5_)_3_(acetone)]	14 *μ*m	5 nm	[14]
[(C_6_H_5_)_3_Sn(MBZT)]	19 *μ*m	1500–3000 nm	[15]
[(C_6_H_5_)_3_Sn(MBZO)]	16 *μ*m	1300–3000 nm	[15]
[(C_6_H_5_)_3_Sn(CMBZT)]	21 *μ*m	500–800 nM	[15]
[(C_6_H_5_)_2_Sn(CMBZT)_2_]	10 *μ*m	300–500 nm	[15]
[(*n*-C_4_H_9_)_2_Sn(CMBZT)_2_]	13 *μ*m	600–800 nm	[15]
[(CH_3_)_2_Sn(CMBZT)_2_]	14 *μ*m	5000–7500 nm	[15]
[(CH_3_)_2_Sn(PMT)_2_]	61 *μ*m	20000–60000 nm	[16]
[(*n*-C_4_H_9_)_2_Sn(PMT)_2_]	26 *μ*m	700 nm	[16]
[(C_6_H_5_)_2_Sn(PMT)_2_]	21 *μ*m	1000–2000 nm	[16]
[(C_6_H_5_)_3_Sn(PMT)]	17 *μ*m	100 nm	[16]

[*]: This work; HTBA: 2-thiobarbituric acid; H_2_MNA: 2-mercapto-nicotinic acid; MBZT: 2-mercapto-benzothiazole; MBZO: 2-mercapto-benzoxazole; CMBZT: 5-chloro-2-mercapto-benzothiazole; HPMT: 2-mercapto-pyrimidine.
